# Hydrogen-exchange behavior of the L20A mutant of the protein A B domain in guanidinium chloride: evidence for persistent native-like contacts

**DOI:** 10.1038/s41598-026-55655-x

**Published:** 2026-06-08

**Authors:** Seiichiro Hayashi, Saeko Yanaka, Maho Yagi-Utsumi, Yukiko Isono, Koichi Kato, Kunihiro Kuwajima

**Affiliations:** 1 Institute for Molecular Science, National Institutes of Natural Sciences, Okazaki, Aichi Japan; 2 Exploratory Research Center on Life and Living Systems, National Institutes of Natural Sciences, Okazaki, Aichi Japan; 3 Core for Spin Life Sciences, Okazaki Collaborative Platform, National Institutes of Natural Sciences, Okazaki, Aichi Japan; 4 Materials and Structures Laboratory at Institute of Integrated Research, Institute of Science Tokyo, Tokyo, Japan; 5 Graduate School of Pharmaceutical Sciences, Nagoya City University, Nagoya, Aichi Japan; 6 SOKENDAI (The Graduate University for Advanced Studies), Okazaki, Aichi Japan; 7 Department of Physics, School of Science, University of Tokyo, Tokyo, Japan

**Keywords:** Biochemistry, Biophysics, Chemistry, Structural biology

## Abstract

Residual structure in unfolded proteins plays a crucial role in directing folding pathways. Here, we examined the unfolded state of the B domain of staphylococcal protein A carrying the L20A mutation, which disrupts native hydrophobic contacts between Leu20 and helix H3 (residues 41–56). The L20A mutant markedly reduced the thermodynamic stability of the native state. Using dimethylsulfoxide (DMSO)-quenched hydrogen/deuterium (H/D)-exchange NMR spectroscopy, we measured H/D-exchange kinetics for backbone amide protons in 6 M guanidinium chloride. At defined exchange times, the reaction mixture was rapidly transferred to a DMSO solution using a spin desalting column, thereby quenching exchange and allowing accurate NMR measurements. Even under the strongly denaturing condition of 6 M guanidinium chloride, the L20A mutant exhibited substantially lower protection against H/D exchange than the wild-type protein, particularly for residues in helix H3. These results indicate that native-like tertiary contacts between Leu20 and helix H3 persist in the unfolded ensemble and contribute to stabilizing residual structure. Our findings demonstrate that DMSO-quenched H/D-exchange NMR combined with targeted mutation provides a powerful approach for dissecting residual interactions and elucidating how early native-like contacts shape folding pathways.

**Supplementary Information:**

The online version contains supplementary material available at 10.1038/s41598-026-55655-x.

## Introduction

The problem of protein folding encompasses two major challenges: (1) predicting the unique three-dimensional structure of a native protein from its amino acid sequence, and (2) elucidating the molecular mechanisms underlying the kinetic folding process from the unfolded state ensemble (hereafter referred to as “the U state”) to the native (N) state. While the first challenge has recently been addressed through remarkable advances in structure-prediction methods powered by state-of-the-art machine learning approaches^1,2^, the second remains unsolved. Elucidating the molecular mechanisms of kinetic folding in globular proteins therefore continues to be a fundamental challenge in biophysics and molecular biology. A mechanistic understanding of folding therefore requires a precise characterization of the U state, which defines the starting conformational landscape for most folding reactions.

Traditionally, the U state of globular proteins unfolded in 6 M guanidinium chloride (GdmCl) or 8 M urea was widely assumed to adopt a random-coil conformation. This assumption, originally based on the scaling relationship characteristic of random-coil chains between intrinsic viscosity and residue number^3^, long served as a cornerstone in protein-folding studies. A similar scaling relationship was also found in the radius of gyration and Stokes radius of a random chain with the residue number^4,5^. Anfinsen’s thermodynamic hypothesis was likewise demonstrated using disulfide-reduced ribonuclease A that refolded spontaneously from an assumed random coil in 8 M urea^6^. The random-search argument in the Levinthal paradox was also predicated on this concept^7,8^.

However, this paradigm has been substantially revised. Numerous multidimensional heteronuclear NMR studies have demonstrated the persistence of significant residual structure in the U state under concentrated denaturant conditions^9,10^. Deviations of backbone chemical shifts from random-coil values, ^3^*J*_HNHα_ coupling constants, residual intensities of heteronuclear single quantum coherence (HSQC) cross peaks, NOE patterns, and ^1^^5^N relaxation data have all been used to detect and characterize residual structure in a variety of proteins, including the N-terminal domain of the phage 434 repressor^11,12^, the FK506-binding protein^13^, barnase^14^, barstar^15^, lysozyme and α-lactalbumin^16–18^, an immunoglobulin superfamily domain Ig18’^19^, the human papillomavirus E2 DNA-binding domain^20^, HIV-1 protease^21^, the chemotactic protein CheY^22^, intestinal fatty acid binding protein^23^, ubiquitin^24^, and cellular retinoic acid-binding protein 1^25^. Importantly, although random-coil scaling behavior is preserved in such systems, satisfying this relationship does not necessarily imply a true random-coil conformation^26,27^. Thus, precise characterization of residual structure in the U state is indispensable for understanding the molecular origins of protein folding.

Modern NMR spectrometers are often equipped with cryogenically cooled probes, which enhance sensitivity but restrict the use of highly saline solutions such as 6 M GdmCl. Consequently, most multidimensional NMR studies of U states have employed the non-ionic denaturant urea. As a denaturant, GdmCl is stronger than urea and is often used in protein-folding studies. To enable NMR analysis under GdmCl conditions, we recently improved the dimethylsulfoxide (DMSO)-quenched hydrogen/deuterium (H/D)-exchange two-dimensional (2D) NMR method originally developed by Zhang et al. (1995)^28^, using spin desalting columns^29–31^. In this improved method, the solvent of the H/D-exchange reaction mixture is rapidly exchanged for a DMSO/D_2_O solution (94.5% DMSO-*d*₆/5.0% D₂O/0.5% dichloroacetic acid (DCA)-*d*_2_, pH* 5.4) within a spin desalting column, thereby quenching the exchange reaction before NMR measurement; pH* indicates the pH-meter reading. Importantly, the H/D-exchange reaction itself is carried out in GdmCl, and only the solvent for NMR detection is replaced with the DMSO/D_2_O solution to suppress further H/D exchange. Because the NMR solvent is replaced prior to measurement, this method imposes no restrictions on the denaturant used during H/D-exchange.

Using this approach, we recently demonstrated the presence of extensive residual structure in the U state of the B domain of staphylococcal protein A (BDPA) in 6 M GdmCl^32^. We determined H/D-exchange protection (*P*) factors for 21 backbone amide protons that form α-helical hydrogen bonds in the native structure. Many of these sites exhibited significant protection, with *P* values between 2.0 and 5.2 even under strongly denaturing conditions. Among the three helices—H1, H2, and H3—helix H3, particularly its C-terminal region, showed the highest protection, with *P* values up to 5.2 for Ala49 and Lys51. These protections were attributed to stabilizing salt bridges between Glu48–Lys51 and Lys50–Asp54^32^.

**Fig. 1:**
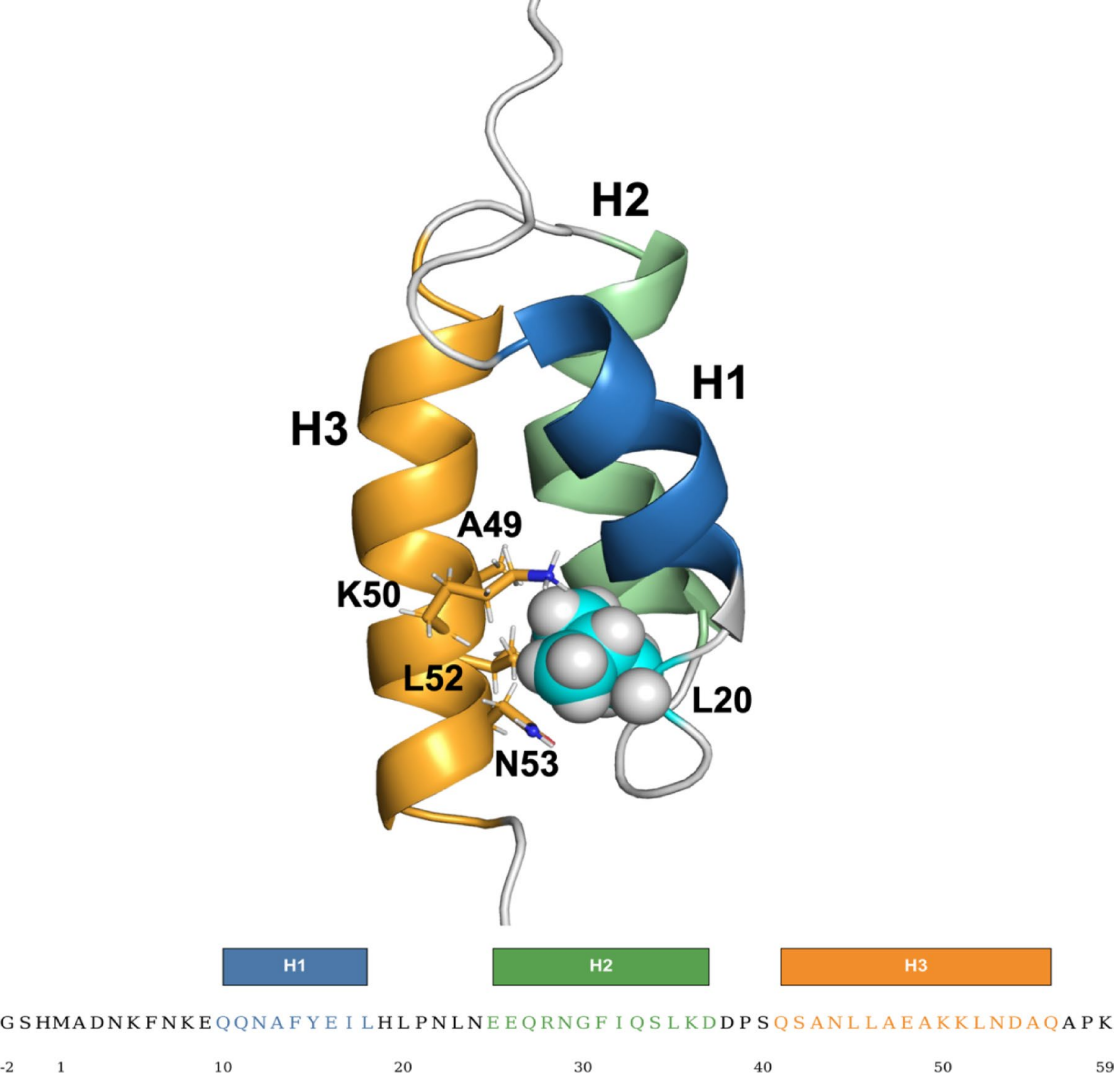
Native three-dimensional structure of BDPA (PDB ID: 1BDD). Leu20 is shown as a cyan space-filling model, whereas Ala49, Lys50, Leu52, and Asn53 are shown as stick models. These residues, located in helix H3, are in close spatial proximity to Leu20 and together form a hydrophobic core that likely contributes to tertiary structure stabilization in the N state. The lower panel shows the amino acid sequence and secondary-structure assignment of BDPA; helices H1, H2, and H3 are colored blue, green, and orange, respectively, and the same color scheme is used throughout the figures. The structure was rendered using PyMOL.

In the native structure of BDPA (PDB ID: 1BDD), the side chains of Leu20 and Leu23 form hydrophobic contacts with residues in the C-terminal portion of helix H3, generating a hydrophobic core between helix H3 and the loop comprising residues 20–23. Notably, the Cδ atoms of Leu20 lie within 5 Å of the side-chain carbons of Ala49, Lys50, Leu52, and Asn53 (Fig. 1). To investigate the role of these native tertiary contacts, we constructed the L20A mutant, thereby abolishing interactions between Leu20 and helix H3.

Here, we characterize the thermodynamic stability and H/D-exchange properties of the L20A mutant in 6 M GdmCl using peptide circular dichroism (CD) spectroscopy and the improved DMSO-quenched H/D-exchange 2D-NMR method. While the mutant retains the native fold under physiological conditions, its thermodynamic stability is markedly reduced. Strikingly, the mutation also substantially destabilizes the residual structure in the U state, as reflected by pronounced decreases in *P* values, particularly in the C-terminal segment of helix H3. These findings demonstrate that native-like tertiary interactions centered on Leu20 are at least partially preserved in the U state of wild-type BDPA, and that their disruption significantly perturbs the unfolded-state energy landscape. More broadly, the results highlight the exceptional sensitivity of DMSO-quenched H/D-exchange 2D-NMR spectroscopy for detecting subtle yet functionally relevant residual structure in unfolded proteins.

## Results

### CD spectra and equilibrium unfolding transitions of wild-type BDPA and the L20A mutant

**Fig. 2:**
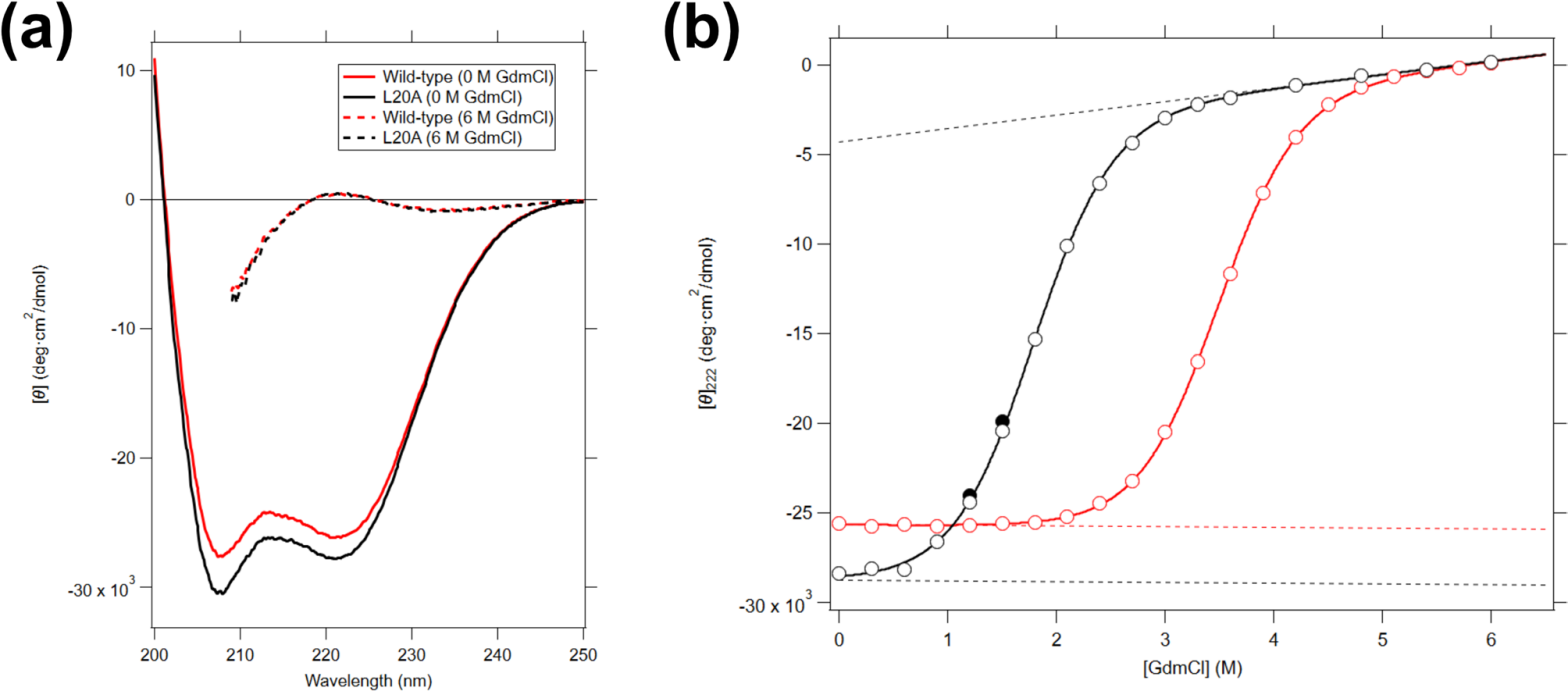
Far-UV CD spectra of L20A (black) and wild-type (WT; red) BDPA at 0.0 and 6.0 M GdmCl (a), and equilibrium unfolding transition curves of WT (red) and L20A (black) monitored by the mean residue ellipticity at 222 nm (b). Measurements were performed in 0.10 M NaCl–20 mM HCOONa buffer in 90% D_2_O/10% H_2_O at pH* 3.4 and 15.0 °C. The solid lines in (b) represent the best fits of the data to Eq. (5). The black and red dashed lines extrapolated from the N state region at low GdmCl concentrations indicate the ellipticity values in the pure N state for WT and L20A, respectively. Open circles show unfolding data obtained by increasing [GdmCl], and filled circles show refolding data obtained after dilution from the unfolding conditions to the indicated [GdmCl]. The ellipticity in the pure U state is assumed to be identical for WT and L20A and represented by the black dashed line extrapolated from the high-GdmCl region.

Figure 2a shows CD spectra of wild-type BDPA and the L20A mutant in the N state at 0 M GdmCl and in the U state at 6.0 M GdmCl, both measured in 90% D_2_O/10% H_2_O at pH* 3.4 and 15.0 °C. In the N state, both proteins exhibit typical α-helical spectra with double minima at 208 and 222 nm. However, the L20A mutant shows increased negative ellipticity at these wavelengths, suggesting an apparent increase in helicity when interpreted solely from the far-UV CD spectrum.

Secondary-structure analysis using SESCA with the 5DS-4 basis set^33,34^ estimated a helix content of 64% for wild-type BDPA, which increased to 71% in the L20A mutant (Figure S1, Table S1). In wild-type BDPA, Leu20 is located in the coil-to-turn region between helix H1 (residues 10–18) and helix H2 (residues 25–37) (Fig. 1). We therefore examined whether the apparent increase in helicity of the mutant reflects extension of adjacent helices using JPred4^35^ and PSIPRED^36^, both of which employ machine-learning-based bioinformatics prediction. However, no helix extension was predicted in the mutant protein. We further applied the Agadir statistical-mechanical algorithm^37^ to estimate residue-specific helical propensity, finding that propensity profiles of the two proteins are largely similar, except that the residues at the N-terminal side of position 20 show slightly lower propensities in the mutant than in the wild-type (Table S2).

We measured near-UV CD spectra of wild-type BDPA and the L20A mutant in both the N and U states (Fig. S2). In the N state, the spectra showed a small but clear difference between 255 and 280 nm, where phenylalanine and tyrosine side chains exhibit CD signals (*L*_b_ transitions) when they are in an asymmetric environment^38^. In the U state, the spectra of the two proteins are nearly identical, indicating that the asymmetric structures of the aromatic side chains are disrupted. Because the aromatic side chains have stronger CD bands in the far-UV region (*L*_a_ transitions), it is likely that these bands contribute to the differences observed in the peptide CD spectra between the wild-type and mutant proteins in the N state (Fig. 2a). In particular, Phe and Tyr side chains have *L*_a_ transitions at approximately 210 and 230 nm, respectively^38^. The N state structures of the two proteins were also examined by 2D ^1^H-^15^N HSQC spectroscopy (Fig. S4a and S4b). The spectra show similar overall dispersion and peak patterns, indicating that the L20A mutant remains folded and retains an overall native-like structure. However, the residue-specific chemical shift differences indicate that native tertiary contacts between residue 20 (leucine in the wild-type) and helix H3 are disrupted in the mutant, as significant perturbations are observed not only near the mutation site but also in residues 48–54 (Fig. S5a). Chemical shift index analysis of the backbone chemical shifts indicates similar helical propensities for wild-type BDPA and the L20A mutant, suggesting that the overall native-state secondary-structure framework is largely preserved in the mutant (Fig. S5b). Taken together, these results suggest that the L20A substitution primarily perturbs native tertiary packing and aromatic side-chain environments, rather than causing a major change in the native-state secondary-structure framework. The apparent discrepancy between the peptide CD spectra (Fig. 2a), which suggest an increase in helicity in the mutant, and the chemical shift index analysis (Fig. S5b) may be explained either by contributions from the aromatic CD bands (*L*_a_transitions) to the peptide region or by differences in the actual molar extinction coefficients of the proteins. Although we used identical extinction coefficients for both proteins, as estimated by the method of Pace et al^39^. (see Materials), the true values in the N state (0 M GdmCl) may differ.

In the U state at 6.0 M GdmCl, the peptide CD spectra of the two proteins are essentially superimposable and characteristic of a fully disordered state. Importantly, this observation does not necessarily imply a completely random-coil conformation. Previous studies^32,40,41^ have shown that the CD intensity of a short helix strongly depends on helix length, and that short, fluctuating helices contribute little to the CD signal, consistent with the spectra observed for the U state in Fig. 2a. Indeed, we have previously demonstrated the presence of residual structures in the U state using H/D-exchange measurements^32^. To further examine whether such residual structure might give rise to detectable differences in CD under strongly denaturing conditions, we monitored the temperature dependence of the mean residue molar ellipticity at 222 nm, [*θ*]_222_ (deg∙cm^2^/dmol), in 6.0 M GdmCl (Fig. S3). Although no distinct cooperative transition was observed, the [*θ*]_222_ values of both proteins gradually decreased with increasing temperature. Such behavior has also been reported for the denaturant-induced U state of many globular proteins^42^. From Fig. S3, wild-type and L20A BDPA exhibit different temperature-dependent slopes, suggesting that their unfolded-state ensembles are not identical under these conditions.

Figure 2b shows the equilibrium unfolding transition curves of wild-type BDPA and the L20A mutant measured by [*θ*]_222_ as a function of GdmCl concentration. The data were analyzed using a two-state unfolding model between the N and U states (Eqs. (3)–(5)). The resulting thermodynamic parameters—the standard free energy change Δ*G*º_NU_ and the cooperativity parameter *m*_NU_—are summarized in Table 1. The midpoint of unfolding (Δ*G*º_NU_/*m*_NU_) shifts from approximately 3.5 M for wild-type BDPA to ~ 1.8 M for the L20A mutant, indicating substantial destabilization of the N state by the mutation. The *m* values, which are proportional to the change in solvent-accessible surface area upon unfolding^43^, are essentially identical between the two proteins (Table 1), suggesting similar global unfolding transitions. To confirm reversibility of unfolding for the L20A mutant, we also measured refolding upon stepwise dilution from the unfolded condition. Filled circles in Fig. 2b indicate the [*θ*]_222_ values of the protein after dilution of GdmCl, and they are well coincident with the unfolding transition curve, indicating the reversibility of the unfolding transition.

**Table 1:** Equilibrium unfolding parameters of the wild-type (WT) and mutant forms of BDPA.

	WT	L20A
Δ*G*º_NU_ (kcal/mol)	5.42 ± 0.08	2.76 ± 0.05
*m*_NU_ (kcal/mol/M)	1.56 ± 0.02	1.56 ± 0.03

### H/D-exchange kinetics of L20A BDPA

H/D-exchange experiments on the L20A mutant were performed under the same conditions as those used previously for wild-type BDPA^32^. The exchange reactions were initiated by ten-fold dilution of 3 mM ^15^N-labeled L20A BDPA at 6.0 M GdmCl/H_2_O into a 6.0 M GdmCl/D_2_O solution at 15.0 °C, yielding a final pH* of 3.2–3.4. At pre-determined exchange time points, the exchange reaction was quenched by rapid medium exchange into the DMSO/D_2_O solution (94.5% DMSO-*d*₆/5.0% D₂O/0.5% DCA-*d*_2_, pH* 5.4) using a spin desalting column (Zeba Spin Desalting Column 89891), followed by measurements of the HSQC spectra. All NMR data were therefore collected in the DMSO/D_2_O solution, whereas the exchange reaction was performed in 6 M GdmCl under the indicated condition.

The H/D-exchange kinetics were monitored by following the decay of backbone amide (NH) peak volumes in two-dimensional^1^H-^15^N HSQC spectra acquired in the DMSO/D_2_O solution as a function of exchange time in 6.0 M GdmCl. The HSQC spectrum of the L20A mutant closely resembles that of wild-type BDPA^32^, except in the vicinity of the mutation site (Figure S6a). Excluding severely overlapped resonances, exchange kinetics were successfully analyzed for 31 residues in the L20A mutant, compared with 29 residues in the wild-type protein.

**Fig. 3:**
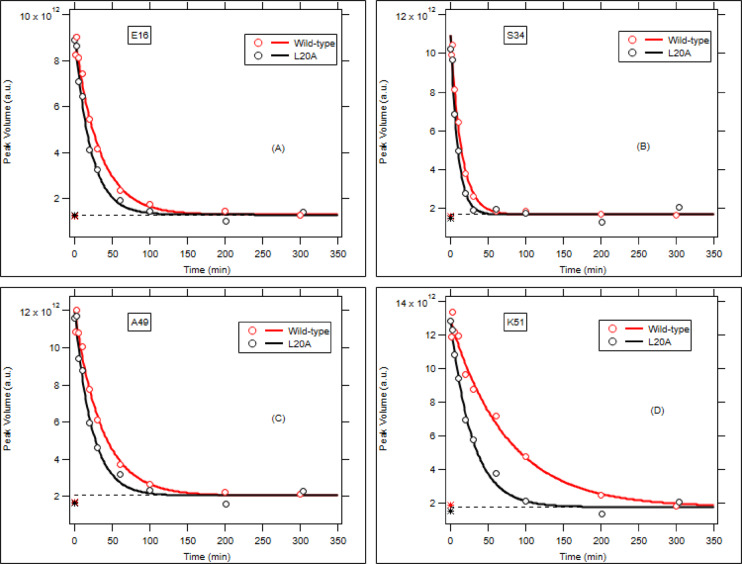
H/D-exchange kinetics for Glu16, Ser34, Ala49, and Lys51 of the L20A mutant (black) and wild-type (WT; red) BDPA in 6.0 M GdmCl at pH* 3.4 and 15.0 °C. The solid lines represent best fits to a single-exponential function (Eq. (7)). In each panel, the dashed line indicates the theoretically estimated peak volume after complete exchange (*Y*(∞) in Eq. (7)), and the asterisk (*) at 0 min denotes the experimentally observed value after heating the samples at 50 °C for 30 min. The wild-type data were taken from the previous study^32^. To facilitate direct comparison, the Δ*Y* and *Y*(∞) parameters in Eq. (7) were numerically adjusted to match between the two proteins. The *k*_obs_ values for the four residues are: Glu16, (4.68 ± 0.32) x 10^− 2^ min^− 1^ (L20A) and (3.10 ± 0.30) x 10^− 2^ min^− 1^ (WT); Ser34, (10.48 ± 0.88) x 10^− 2^ min^− 1^ (L20A) and (7.04 ± 0.64) x 10^− 2^ min^− 1^ (WT); Ala49, (4.37 ± 0.41) x 10^− 2^ min^− 1^ (L20A) and (2.78 ± 0.29) x 10^− 2^ min^− 1^ (WT); and Lys51, (3.45 ± 0.24) x 10^− 2^ min^− 1^ (L20A) and (1.33 ± 0.16) x 10^− 2^ min^− 1^ (WT), where the errors are fitting error estimates.

All observed exchange decays were well described by a single-exponential function (Eq. (7)), yielding the observed exchange rate constant *k*_obs_. Figure 3 shows representative H/D-exchange curves for Glu16, Ser34, Ala49 and Lys51, compared directly with the corresponding curves of wild-type BDPA. For all four residues, exchange is significantly accelerated in the L20A mutant, although the magnitude of the effect varies among residues (Fig. 3).

### Protection factor

The protection factor (*P*), which quantifies the degree to which an NH group is protected from exchange with solvent deuteron, is defined as the ratio of the intrinsic exchange rate constant (*k*_int_) to *k*_obs_ :

1 $$P = \frac{k_{\mathrm{int}}}{k_{\mathrm{obs}}}$$

The value of *k*_int_ depends on solution conditions (pH*, GdmCl concentration, and temperature) as well as primary structure effects from neighboring residues. We previously determined the catalytic constants for specific acid (*A*), specific base (*B*), and water (*C*) catalysis (see Eq. (8)) from the pH* dependence of H/D-exchange in poly-DL alanine under identical conditions (6.0 M GdmCl and 15.0 °C)^32^. Primary-structure correction factors (*A*_L_, *A*_R_, *B*_L_ and *B*_R_) reported by Bai et al. (1993)^44^ and Nguyen et al. (2018)^45^ allow accurate estimation of *k*_int_ for individual NH groups.

For NH groups fully exposed to solvent, such as in random-coil poly-DL-alanine, *P* = 1. In folded N state proteins, the most strongly protected NH protons often exhibit *P* > 10^5^, corresponding to opening free energy exceeding ~ 6 kcal/mol^46^, whereas molten globule intermediates typically show *P* values in the range of 10^2^ − 10^3^^47,48^.

**Fig. 4:**
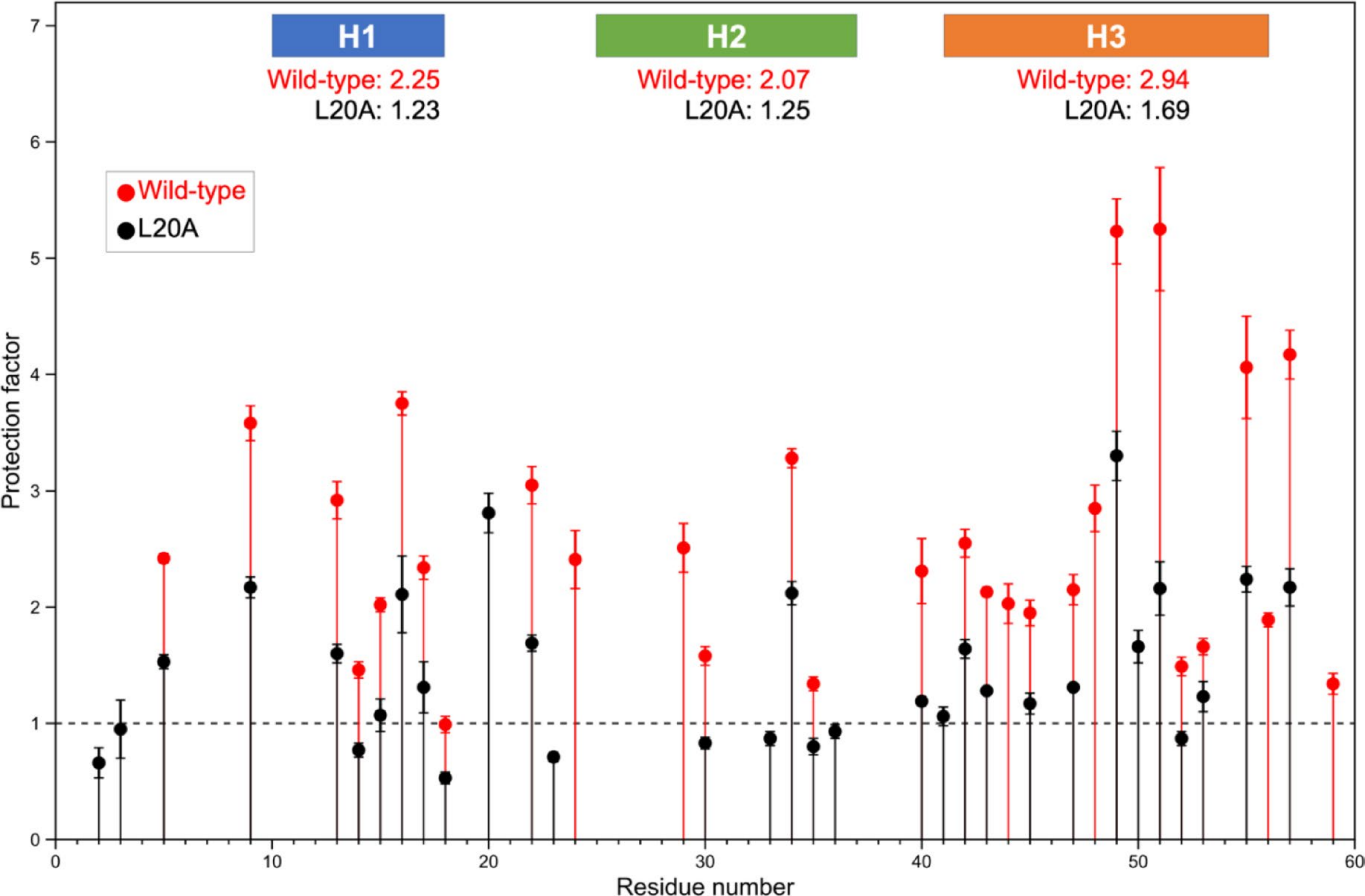
Residue-wise distribution of H/D-exchange *P* values for the L20A mutant (black) and wild-type BDPA (red) as a function of residue number, measured at pH 3.2–3.4 in 6.0 M GdmCl at 15.0 °C. The *P* values were obtained from 3–4 independent experiments, and error bars indicate standard errors. The vertical lines connect each value to the x-axis for clarity. The dashed horizontal line indicates *P* = 1. Colored boxes denote helices H1, H2, and H3, colored in the same manner as in Fig. 1. The values shown beneath each box are the mean *P* values calculated from residues within the corresponding helix for which the *P* values were obtained for both wild-type and L20A. The L20A mutant exhibits significantly lower *P* values than the wild-type protein across the entire sequence.

Table S3 summarizes the *P* values obtained for wild-type and L20A mutant BDPA under the present denaturing condition (6.0 M GdmCl, pH* 3.2–3.4, 15 °C); the data for the wild-type protein were taken from the previous study^32^. Figure 4 compares the residue-level distributions of *P* values between the two proteins. Across all analyzed residues, the L20A mutant consistently exhibits lower *P* values than wild-type BDPA. Notably, residues distant from the mutation site in the primary sequence —such as Ala49 and Lys51—show marked reduction in protection. Specifically, the *P* values of Ala49 and Lys51 decrease from 5.2 in wild-type BDPA to 3.3 and 2.2, respectively, in the L20A mutant. Structural mapping of the *P* values onto the native BDPA structure also highlights that the reduction in protection in the L20A mutant is pronounced in the C-terminal region of helix H3 (Fig. S7).

These reductions strongly suggest that the L20A mutation destabilizes residual tertiary interactions that persist in the U state under strongly denaturing conditions. Because H/D-exchange protection reflects hydrogen-bond formation between an NH group and an acceptor such as a peptide carbonyl, the fraction of hydrogen-bond formation (*f*_Hbond_) is related to *P* as^32^:

2 $$f_{\mathrm{Hbond}} = 1 - \frac{1}{P}$$

Thus, NH groups with *P* = 5.2 correspond to *f*_Hbond_ ≈ 0.81, whereas reductions to *P* = 3.3 and 2.2 decrease *f*_Hbond_ to approximately 0.69 and 0.54, respectively. These results indicate that the L20A mutation weakens hydrogen bonding throughout the protein, consistent with a global destabilization of residual structure in the U state.

## Discussion

In this study, we investigated (1) the equilibrium unfolding behavior of wild-type and L20A mutant BDPA using peptide CD spectroscopy (Fig. 2), and (2) the H/D-exchange behavior of the L20A mutant in the U state at 6.0 M GdmCl (pH 3.2–3.4 and 15 °C) using DMSO-quenched H/D-exchange 2D-NMR spectroscopy with spin desalting columns. The H/D-exchange data were compared with those previously obtained for wild-type BDPA (Fig. 4). The L20A mutation markedly reduced the thermodynamic stability of the protein, shifting the unfolding midpoint from ~ 3.5 M to ~ 1.8 M GdmCl (Fig. 2). Importantly, the mutation also destabilized the residual structure retained in the U state, as reflected by substantially reduced H/D-exchange *P* values. Traditionally, proteins unfolded in 6 M GdmCl or 8 M urea were considered to adopt random-coil conformations^3^. This view was largely based on the scaling relationship between intrinsic viscosity and chain length characteristic of random polymers^3–5^. Because conventional spectroscopic techniques such as CD, optical rotatory dispersion, and aromatic absorption lacked the sensitivity to detect non-random features, the U state was long assumed to be devoid of structure. Subsequent multidimensional NMR studies, however, revealed that many proteins retain significant residual structure even under strongly denaturing conditions^49^. Identifying the interactions that stabilize such residual structure is thus essential for elucidating the molecular determinants of protein folding. Below, we discuss: (1) the interactions responsible for stabilizing residual structure in the U state; (2) DMSO-quenched H/D-exchange 2D-NMR spectroscopy as a sensitive tool for characterizing residual structure in unfolded proteins; and (3) implications of our findings for the folding mechanisms of BDPA and its L20A mutant.

Our previous study suggested that the formation of two salt-bridges, i.e., those of Glu48–Lys51 and Lys50–Asp54, contributes substantially to stabilizing residual helix H3, yielding protection factors as high as *P* ≈ 5.2 for Ala49 and Lys51^32^. These electrostatic interactions appear to promote partial formation of helix H3 even in the U state. On the other hand, in the native structure of BDPA (PDB ID: 1BDD), the Leu20 side chain forms hydrophobic contacts with Ala49, Lys50, Leu52, and Asn53, creating a hydrophobic cluster between Leu20 and the C-terminal half of helix H3 (Fig. 1). To determine whether these sequence-distant interactions persist in the U state, we analyzed the L20A mutant, which lacks these hydrophobic contacts. The mutation markedly destabilized the residual structure, as reflected by substantial reductions in *P* values across the protein (Fig. 4). The largest effects were observed in the C-terminal half of helix H3, where the *P* values for Ala49 and Lys51 decreased from 5.2 in the wild type to 3.3 and 2.2, respectively, in the mutant (Fig. 4 and Table S3). These results demonstrate that native-like tertiary interactions between Leu20 and helix H3 are at least partially retained in 6 M GdmCl. The fact that Ala49 and Lys51 in the L20A mutant still show protection factors above unity further suggests that local salt-bridge interactions continue to contribute to residual structural stabilization. Finally, sequence-distant long-range interactions that persist between Leu20 and helix H3 in the U state are likely to play a key role in initiating productive refolding upon denaturant dilution. Similar long-range interactions in U states have been identified in other proteins. For example, a single-point mutation in lysozyme disrupted an extensive network of non-local interactions, highlighting their cooperative nature^50^. In cellular retinoic acid-binding protein 1, chemical-shift perturbations upon single-residue substitutions revealed multiple non-local contacts at topologically critical sites for the native fold in the U state^25^. Long-range interactions have also been inferred from deviations from random-coil behavior in the kinetics and equilibrium of histidine–heme loop formation, although this method is limited to heme proteins^9,51^.

DMSO-quenched H/D-exchange 2D-NMR spectroscopy provides exceptional sensitivity for detecting residual structures in unfolded proteins. The use of spin desalting columns enables rapid solvent exchange and quenching within ~ 1 min^29–32^, allowing residue-specific detection of protected NH protons. The present results thus illustrate that the H/D-exchange protection factor (*P*) of a peptide NH group is remarkably sensitive to features of residual structure. Combining this method with site-directed mutagenesis provides a powerful strategy for dissecting cooperative networks of residual interactions with residue-level resolution. DMSO-quenched H/D-exchange 2D-NMR spectroscopy with spin desalting columns is thus a simple yet powerful approach for elucidating relationships between the residual structure and folding mechanisms in globular proteins.

There are two types of folding behavior in globular proteins: (1) two-state folding, in which the folding proceeds directly from U to N without the accumulation of intermediates, and (2) non-two-state folding, in which a stable folding intermediate accumulates along the folding pathway^52,53^. BDPA was initially proposed to follow a two-state mechanism^54–56^, but subsequent time-resolved IR studies revealed a kinetic intermediate in which helices H1 and H3 are partially formed within 100 ns^57,58^. The long-range interactions between Leu20 and helix H3 that persist in the U state likely stabilize this intermediate, guiding BDPA along a productive folding pathway consistent with the framework model of the non-two-state folding^59^. By contrast, the U state of the L20A mutant is more extensively unfolded as indicated by lower *P* values for all residues observed (Fig. 4), suggesting that the interactions stabilizing the kinetic folding intermediate of wild-type BDPA are much weaker in the mutant. It is thus expected that the folding mechanism of the L20A mutant may be much closer to the two-state type. Mutation-induced switching between three-state and two-state folding mechanisms has been observed for several proteins^60–63^. Although a modest change in the folding pathway associated with altered native state packing cannot be excluded, the L20A mutation in BDPA may likewise influence the balance between these folding behaviors. Finally, BDPA, a canonical three-helix bundle protein, is widely used as a model for computational folding studies. All-atom molecular dynamics (MD) simulations, employing diverse methodologies—including Go model with discontinuous potentials^64^, replica exchange^65^^[,66^, integrating tempering sampling^67^, and temperature-aided cascade MD^68^—have successfully reproduced the full folding process from unfolded conformations to the N state. More recently, Pereira and Martinez^69^ applied structure-based models and atomistic simulations to investigate how BDPA folding is coupled to the formation of secondary structure. These simulations consistently identify helix H3 as the earliest and most stable structural element during folding, fully consistent with our experimental observations.

## Conclusions

- Using DMSO-quenched H/D-exchange 2D-NMR spectroscopy, we investigated the H/D-exchange behavior of peptide NH groups in the GdmCl-unfolded state of the L20A mutant of BDPA and compared it with that of the wild-type protein, which exhibits substantial residual structure in 6 M GdmCl, with *P* values reaching ~ 5. The mutation destabilized this residual structure in the U state, as evidenced by marked decreases of the H/D-exchange *P* values. This effect was most pronounced in the C-terminal half of helix H3 but was also observed throughout the mutant (Fig. 4). In the native structure, the Leu20 side chain forms long-range hydrophobic contacts with the C-terminal half of helix H3 (Fig. 1), indicating that the native-like tertiary interactions between Leu20 and helix H3 are at least partly preserved even in the U state.
- DMSO-quenched H/D-exchange 2D-NMR spectroscopy provides exceptional sensitivity for detecting residual structures in unfolded proteins. The use of spin desalting columns enabled rapid quenching of the H/D-exchange reaction and subsequent 2D-NMR measurements, allowing site-specific characterization that conventional methods such as peptide CD could not achieve. This approach not only revealed residual structure in unfolded BDPA but also quantified differences in hydrogen bonding between the wild-type and the L20A mutant. Coupling this technique with site-directed mutagenesis provides a powerful strategy to dissect cooperative networks of residual interactions at residue-level resolution.
- Wild-type BDPA has been reported to fold through a non-two-state pathway in which a kinetic folding intermediate with partially formed helices H1 and H3 accumulates early. The persistence of residual structure in the U state likely stabilizes this intermediate. By contrast, folding of the L20A mutant, in which such structure is largely disrupted (Fig. 4), appears to follow a mechanism much closer to the two-state model. Thus, probing residual structure in unfolded proteins is essential for elucidating the molecular principles that govern protein folding.

## Materials and methods

### Materials

DMSO-*d*_6_ and D_2_O were purchased from Cambridge Isotope Laboratories (Tewksbury, MA, USA). DCA-*d*_2_ was obtained from Sigma–Aldrich (St. Louis, MO, USA) and used as a buffering agent in the quenching DMSO/D_2_O solution (94.5% [v/v] DMSO-*d*_6_/0.5% [v/v] DCA-*d*_2_/5% [v/v] D_2_O, pH* 5.4). GdmCl was purchased from Nacalai Tesque (Kyoto, Japan). Deuterated GdmCl was prepared by repeated cycles of dissolution in D_2_O followed by lyophilization.

Uniformly ^15^N-labeled and uniformly ^13^C, ^15^N-labeled proteins for heteronuclear NMR experiments were expressed in *Escherichia coli* BL21(DE3) cells (SMOBIO Technology Inc., Hsinchu, Taiwan) grown at 37 °C in M9 minimal medium. For uniform ^15^N labeling, ^15^NH_4_Cl was used as the sole nitrogen source. For uniforM ^13^C, ^15^N labeling, ^15^NH_4_Cl and U-^13^C glucose were used as the sole nitrogen and carbon sources, respectively. In addition, ^13^C-leucine/^15^N-alanine selectively labeled wild-type and L20A mutant proteins were prepared for assignment of alanine NH signals in the ^1^H-^15^N HSQC spectrum of the L20A mutant protein (Fig. S6b). Non-labeled proteins were expressed in LB broth and purified as recombinant proteins as described previously^32^.

Protein concentrations were determined spectrophotometrically using a microvolume spectrophotometer (NanoDrop One) with molar extinction coefficients of 1490 M^− 1^ cm^− 1^ at 0 M GdmCl and 1285 M^− 1^ cm^− 1^ at 6.0 M GdmCl at 280 nm^39^.

### Circular dichroism (CD) spectroscopy

CD measurements were performed at 15.0 °C using a Jasco J-1500 spectropolarimeter equipped with a Peltier-type temperature controller. CD spectra were recorded at protein concentrations of 50–70 µM at 15.0 °C using a 0.10-cm path-length cuvette for the far-UV region (< 250 nm) and a 1.00-cm path-length cuvette for the near-UV region (> 250 nm). For the temperature-dependent experiment in 6.0 M GdmCl, the ellipticity at 222 nm was additionally monitored during heating from 10.0 to 50.0 °C using the same samples after spectral acquisition.

Equilibrium unfolding of the L20A mutant was monitored by the mean residue ellipticity at 222 nm (*θ*_obs_) as a function of GdmCl concentration ([GdmCl]) and compared with that of wild-type BDPA (Fig. 2b). Measurements were conducted in 0.10 M NaCl and 20 mM sodium formate buffer in 90% D_2_O/10% H_2_O at pH* 3.4 and 15.0 °C.

Secondary structure content in the N state of wild-type and L20A mutant BDPA was estimated from CD spectra using SESCA version 0.97 with the 5DS-4 basis set^33^^,34^. The results are summarized in Figure S1 and Table S1

The unfolding transition curves of both proteins were well described by a reversible two-state model between N and U as:

3 $$\mathrm{N} \stackrel{K_{\mathrm{NU}}}{\rightleftharpoons} \mathrm{U}$$

where *K*_NU_ is the equilibrium constant. The standard Gibbs free-energy change of unfolding, Δ*G*_NU_, is assumed to depend linearly on [GdmCl]:

4 $$\Delta G_{\mathrm{NU}} = -RT\ln K_{\mathrm{NU}} = \Delta G^\circ_{\mathrm{NU}} - m_{\mathrm{NU}}[\mathrm{GdmCl}]$$

where *R* and *T* are the gas constant and the absolute temperature, respectively, Δ*G*°_NU_ is the Δ*G*_NU_ at [GdmCl] = 0, and *m*_NU_ is the dependence of Δ*G*_NU_on [GdmCl]. The equilibrium unfolding curves were fitted by nonlinear least-squares analysis to the following Eq^70^.:

5 $$\theta_{\mathrm{obs}} = \frac{k_0 + k_1[\mathrm{GdmCl}] + \left(k_2 + k_3[\mathrm{GdmCl}]\right)\exp\left(\frac{-\Delta G^\circ_{\mathrm{NU}} + m_{\mathrm{NU}}[\mathrm{GdmCl}]}{RT}\right)}{1 + \exp\left(\frac{-\Delta G^\circ_{\mathrm{NU}} + m_{\mathrm{NU}}[\mathrm{GdmCl}]}{RT}\right)}$$

where (*k*_0_ + *k*_1_[GdmCl]) and (*k*_2_ + *k*_3_[GdmCl]) correspond to *θ*_obs_ values of the pure N and U states, respectively. The parameter *k*_1_ was constrained to be identical for wild-type and L20A mutant proteins, and the *k*_2_ and *k*_3_ values are also common to the two proteins.

### NMR spectroscopy

All NMR experiments were performed on a Bruker AVANCE-800 spectrometer equipped with a cryogenically cooled TCI probe. Backbone resonance assignments of the ^1^H-^15^N HSQC spectrum of ^15^N-labeled L20A BDPA in DMSO/H_2_O solution (94.5% [v/v] DMSO-*d*_6_/0.5% [v/v] DCA-*d*_2_/5.0% [v/v] H_2_O, pH* 5.4) at 25 °C were obtained by comparison with previously assigned spectra of wild-type BDPA^32^, as most backbone cross-peaks were coincident between the two proteins (Fig. S6a). To achieve the complete assignment, ^13^C-leucine/^15^N-alanine selectively labeled wild-type and L20A mutant proteins were prepared, and ^1^H-^15^N HSQC and HNCO NMR experiments were conducted.

For H/D-exchange experiments, ^1^H-^15^N HSQC spectra of ^15^N-labeled L20A BDPA in the DMSO/D_2_O solution were acquired at 25 °C with 80 (*t*_1_) × 1024 (*t*_2_) complex points and 8 scans per *t*_1_ increment. Data were processed using TopSpin (Bruker) and analyzed with NMRPipe and POKY^71^^,72^.

For native-state spectral comparison, ^1^H-^15^N HSQC spectra of wild-type and L20A BDPA were acquired in 0.10 M NaCl and 20 mM sodium formate buffer in 10% D_2_O at pH 3.4 at 15.0 °C with 512 (*t*_1_) × 1024 (*t*_2_) complex points and 8 scans per *t*_1_ increment. Backbone amide assignments of wild-type and L20A BDPA in the N state were determined from HNCA, HNCOCA, HNCACB, HNCOCACB, HNCO and HNCACO NMR experiments. Chemical shift perturbations between wild-type and L20A were calculated using the following equation.

6 $$\Delta\delta = \sqrt{\left(\Delta\delta_{\mathrm{H}}\right)^2 + \frac{\left(\Delta\delta_{\mathrm{N}}\right)^2}{5}}$$

where Δ*δ*_H_ and Δ*δ*_N_ are the chemical shift differences in the H and N dimensions, respectively. To calculate chemical shift index of wild-type and L20A, predicted random-coil chemical shifts were calculated from the amino acid sequence using the random-coil chemical shift calculator at 15 °C and pH 3.4^73^.

### DMSO-quenched hydrogen/deuterium (H/D) exchange experiments

H/D-exchange was initiated by 10-fold dilution of 3 mM ^15^N-labeled L20A BDPA unfolded in 6.0 M GdmCl–0.10 M NaCl in H_2_O (pH 3.6) into 6.0 M deuterated GdmCl–0.10 M NaCl–11.1 mM sodium formate in D_2_O at pH* 3.2–3.4 and 15.0 °C. The pH* of the reaction mixture was 3.2–3.4 at room temperature (pD 3.6–3.8).

At pre-determined time points, 1.0 mL aliquots were withdrawn, rapidly frozen in liquid nitrogen, and stored at − 80 °C. Prior to NMR analysis, samples were thawed at 7 °C, and the denaturant solution was replaced with the quenching DMSO/D_2_O solution using a spin desalting column (Zeba Spin Desalting Column 89891; Thermo Fisher Scientific K.K., Tokyo) at 20 °C, as described previously^29–31^^,32^.

### Analysis of the H/D-exchange kinetics and the protection factors

H/D-exchange kinetics were analyzed by fitting NMR signal intensity *Y*(*t*) as a function of H/D-exchange time *t* to a single-exponential decay:

7 $$Y(t) = \Delta Y\,\exp\left(-k_{\mathrm{obs}}t\right) + Y(\infty)$$

where Δ*Y*, *k*_obs_, and *Y*(∞) represent the kinetic amplitude, observed exchange rate constant, and final signal intensity, respectively. Four independent H/D-exchange experiments were conducted, and each dataset was fitted separately.

Protection factors (*P*) were calculated as the ratio of the intrinsic exchange rate (*k*_int_) to the observed exchange rate (*k*_obs_) (Eq. (1)). The *k*_int_ values were calculated as described previously^32^:

8 $$k_{\mathrm{int}} = A_{\mathrm{L}}\cdot A_{\mathrm{R}}\cdot A\cdot [\mathrm{D}^{+}] + B_{\mathrm{L}}\cdot B_{\mathrm{R}}\cdot B\cdot [\mathrm{OD}^{-}] + B_{\mathrm{L}}\cdot B_{\mathrm{R}}\cdot C$$

where *A*, *B*, and *C* denote catalytic constants for acid, base, and water catalysis, respectively. The values under the present conditions (6.0 M GdmCl, 15.0 °C) were taken from Yanaka et al. (2023)^32^. Side-chain correction factors (*A*_L_, *A*_R_, *B*_L_, and *B*_R_) were adopted from Bai et al. (1993)^44^ and slightly improved by Nguyen et al. (2018)^45^. Final *P* values are reported as means ± standard errors from the 3–4 independent experiments.

## Supplementary Information

Below is the link to the electronic supplementary material.

Supplementary Material 1

## Data Availability

Assigned chemical shift data for wild-type and L20A BDPA were deposited in the BMRB under the accession numbers 53756 and 53757, respectively.
