# Supplementary material for: Hydrogen-exchange behavior of the L20A mutant of the protein A B domain in guanidinium chloride: evidence for persistent native-like contacts
Source: Sci Rep. 2026 Jun 8;16:25491. doi: 10.1038/s41598-026-55655-x (PMC13476237; doi:10.1038/s41598-026-55655-x)
Supplement: Supplementary file 1 [file 41598_2026_55655_MOESM1_ESM.pdf]

Supplementary Information to

Hydrogen-Exchange Behavior of the L20A Mutant of the Protein A B Domain in Guanidinium Chloride: Evidence for Persistent Native-like Contacts

Seiichiro Hayashi[1], Saeko Yanaka[1 2 3 4], Maho Utsumi Yagi[1 2 3 5], Yukiko Isono[1 2], Koichi Kato[1 2 3 5 6*], Kunihiro Kuwajima[1 6 7 *]

1 Institute for Molecular Science, National Institutes of Natural Sciences, Okazaki, Aichi, Japan

2 Exploratory Research Center on Life and Living Systems, National Institutes of Natural Sciences, Okazaki, Aichi, Japan

3 Core for Spin Life Sciences, Okazaki Collaborative Platform, National Institutes of Natural Sciences, Okazaki, Aichi, Japan

4 Materials and Structures Laboratory at Institute of Integrated Research, Institute of Science Tokyo, Tokyo, Japan

5 Graduate School of Pharmaceutical Sciences, Nagoya City University, Nagoya, Aichi, Japan

6 SOKENDAI (The Graduate University for Advanced Studies), Okazaki, Aichi, Japan

7 Department of Physics, School of Science, University of Tokyo, Tokyo, Japan

Correspondence: Correspondence and requests for materials should be addressed to Koichi Kato (kkatonmr@ims.ac.jp) or Kunihiro Kuwajima (kuwajima@ims.ac.jp)

21

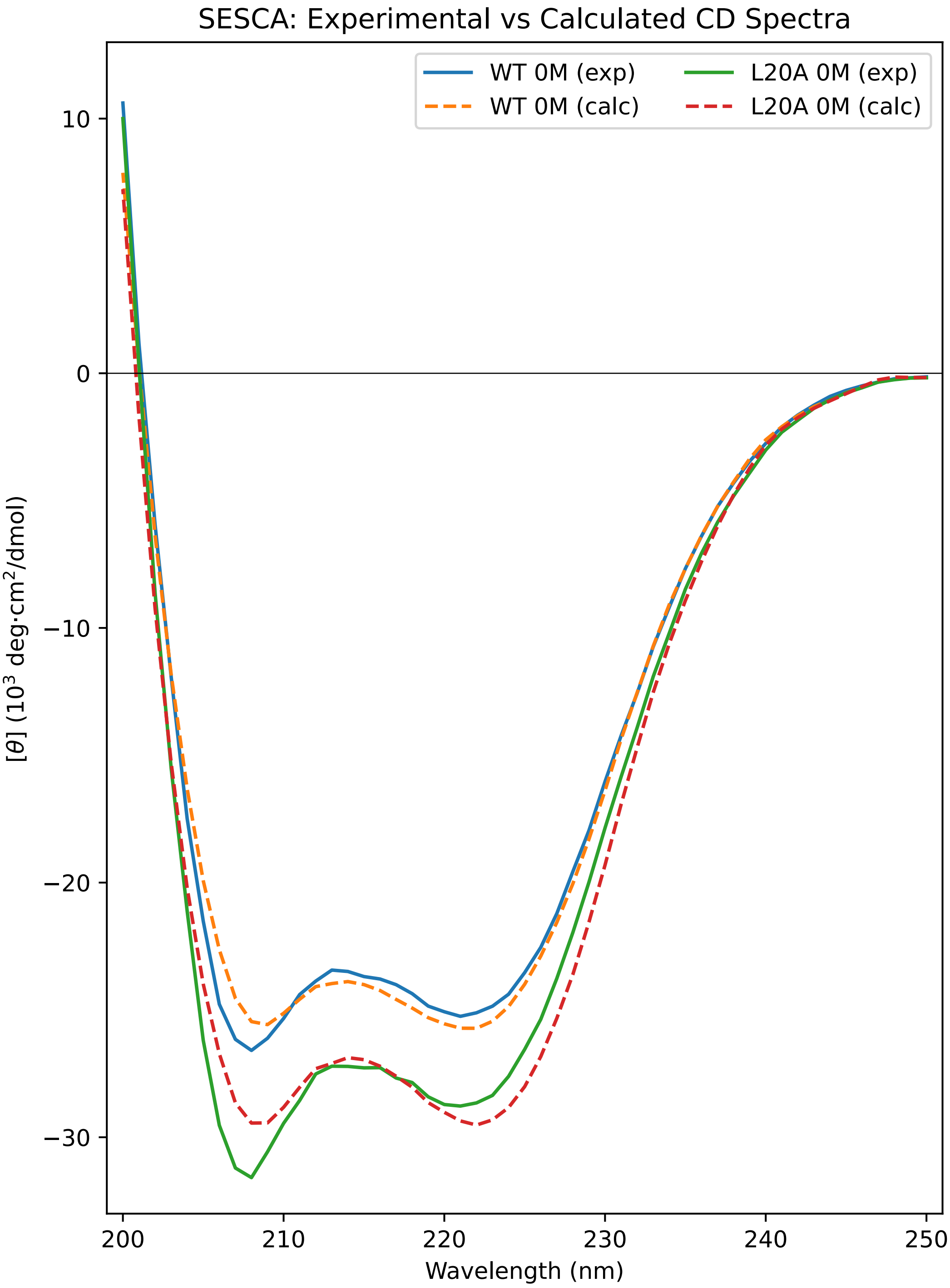

Supplementary Figure S1.

Comparison of experimental and SESCA-calculated CD spectra of wild-type (WT) and L20A mutant BDPA in the N state at 15.0 ºC and 0 M GdmCl (0.10 M NaCl–20 mM HCOONa buffer in 90% D_2O/10% H_2O, pH* 3.4). Solid and dashed lines represent the experimental spectra and SESCA-calculated spectra, respectively, using the 5DS-4 basis set [1,2].

Supplementary Table S1.

	Helix1	Beta1	Helix2	Turn1	Turn2	Other	RMSD (10^3 deg·cm2/dmol)
WT (0 M GdmCl)	0.5941	0.0000	0.0457	0.0000	0.1497	0.2107	0.705
L20A (0 M GdmCl)	0.6897	0.0000	0.0168	0.0000	0.1709	0.1226	1.080

Secondary-structure composition of wild-type (WT) and L20A mutant BDPA in the N state at 15.0 ºC and 0 M GdmCl (0.10 M NaCl–20 mM HCOONa buffer in 90% D_2O/10% H_2O, pH* 3.4) estimated by SESCA version 0.97 using the 5DS-4 basis set [1,2]. Values indicate fractional content.

(a)

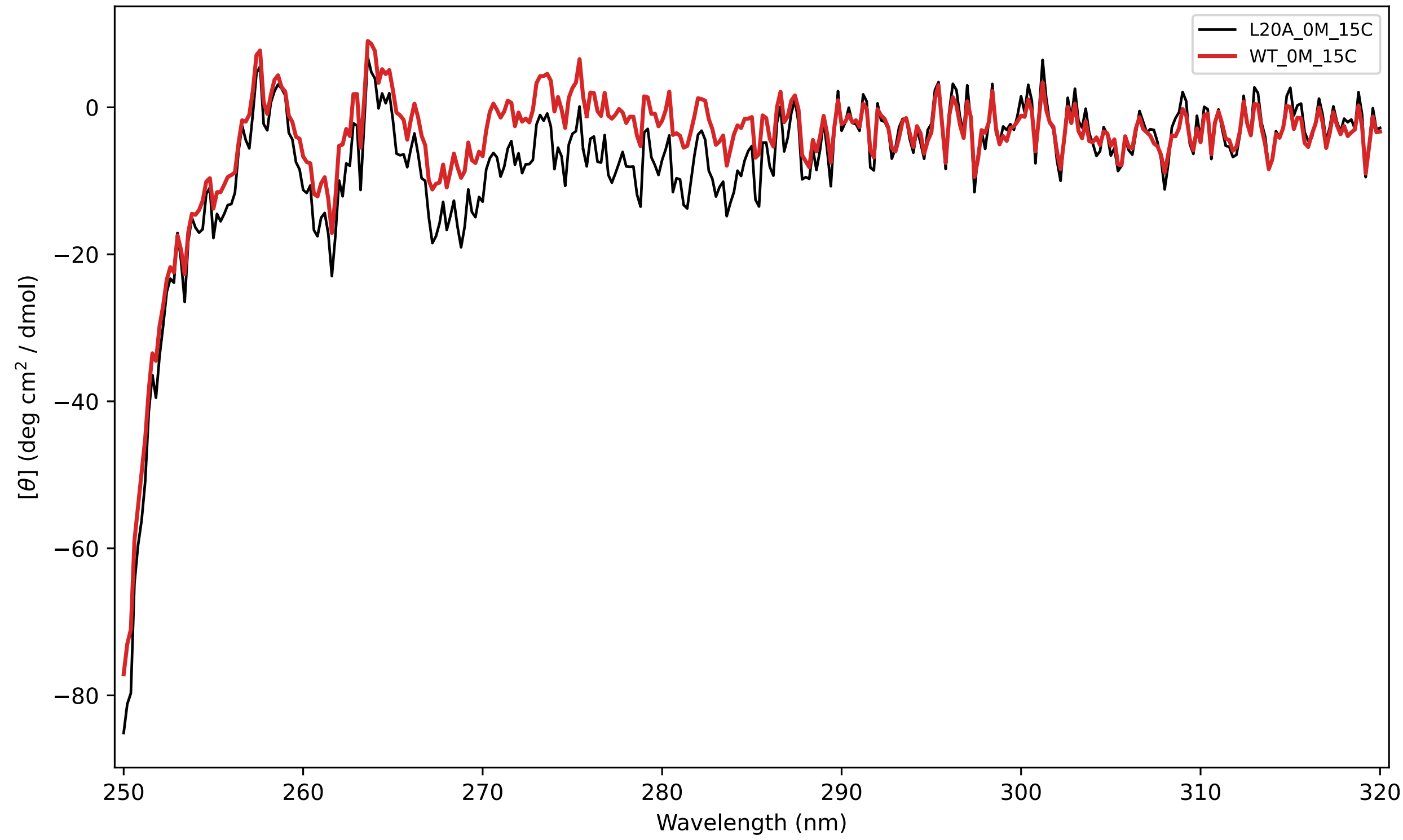
L20A_0M_15C
WT_0M_15C
[θ] (deg cm² / dmol)
0
−20
−40
−60
−80
250
260
270
280
290
300
310
320
Wavelength (nm)

(b)

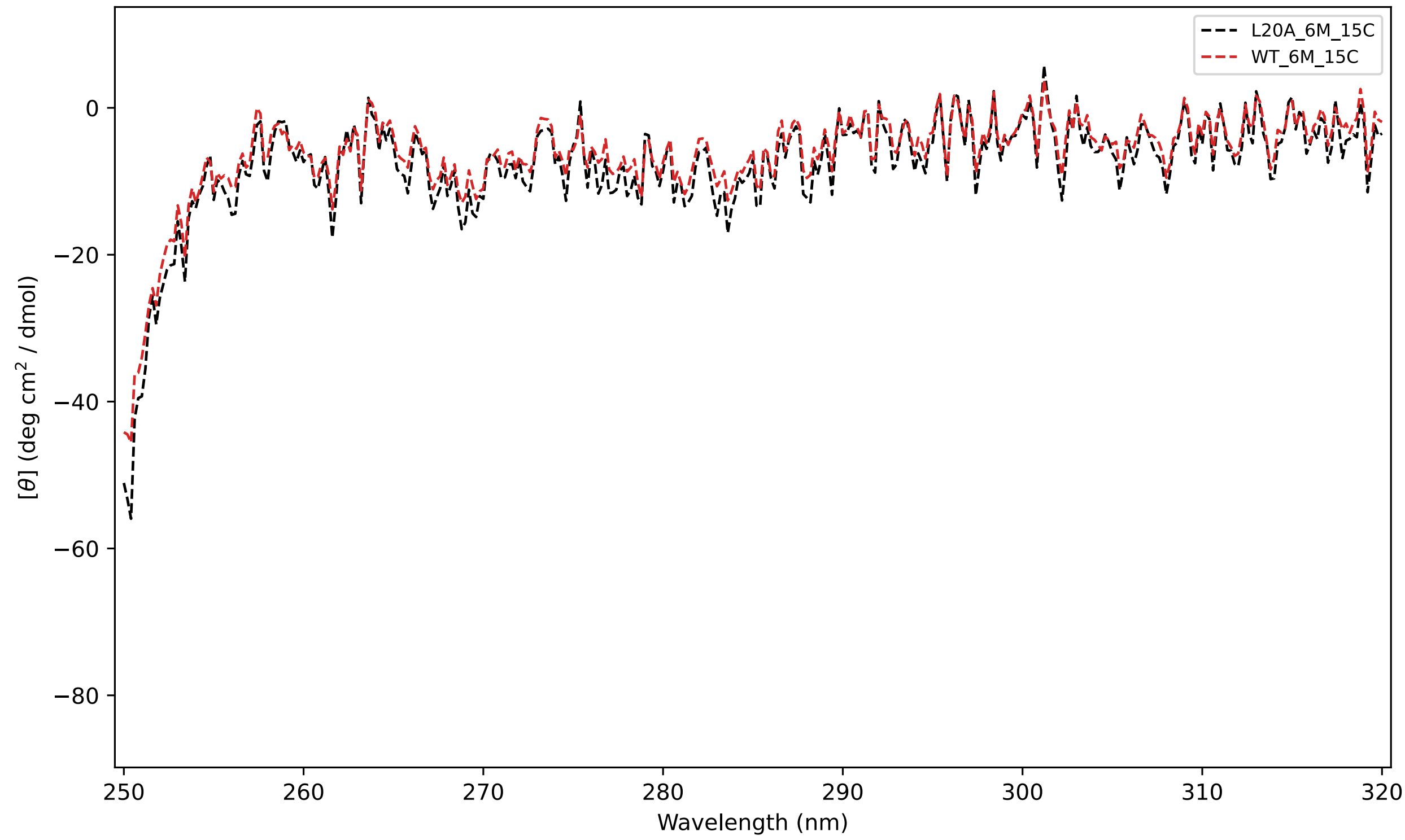
L20A_6M_15C
WT_6M_15C
[θ] (deg cm² / dmol)
0
−20
−40
−60
−80
250
260
270
280
290
300
310
320
Wavelength (nm)

Supplementary Figure S2.

Near-UV CD spectra of wild-type (WT; black) and L20A (red) BDPA at (a) 0.0 and (b) 6.0 M GdmCl. Measurements were performed in 0.10 M NaCl–20 mM HCOONa buffer in 90% D_2O/10% H_2O at pH* 3.4 and 15.0 °C.

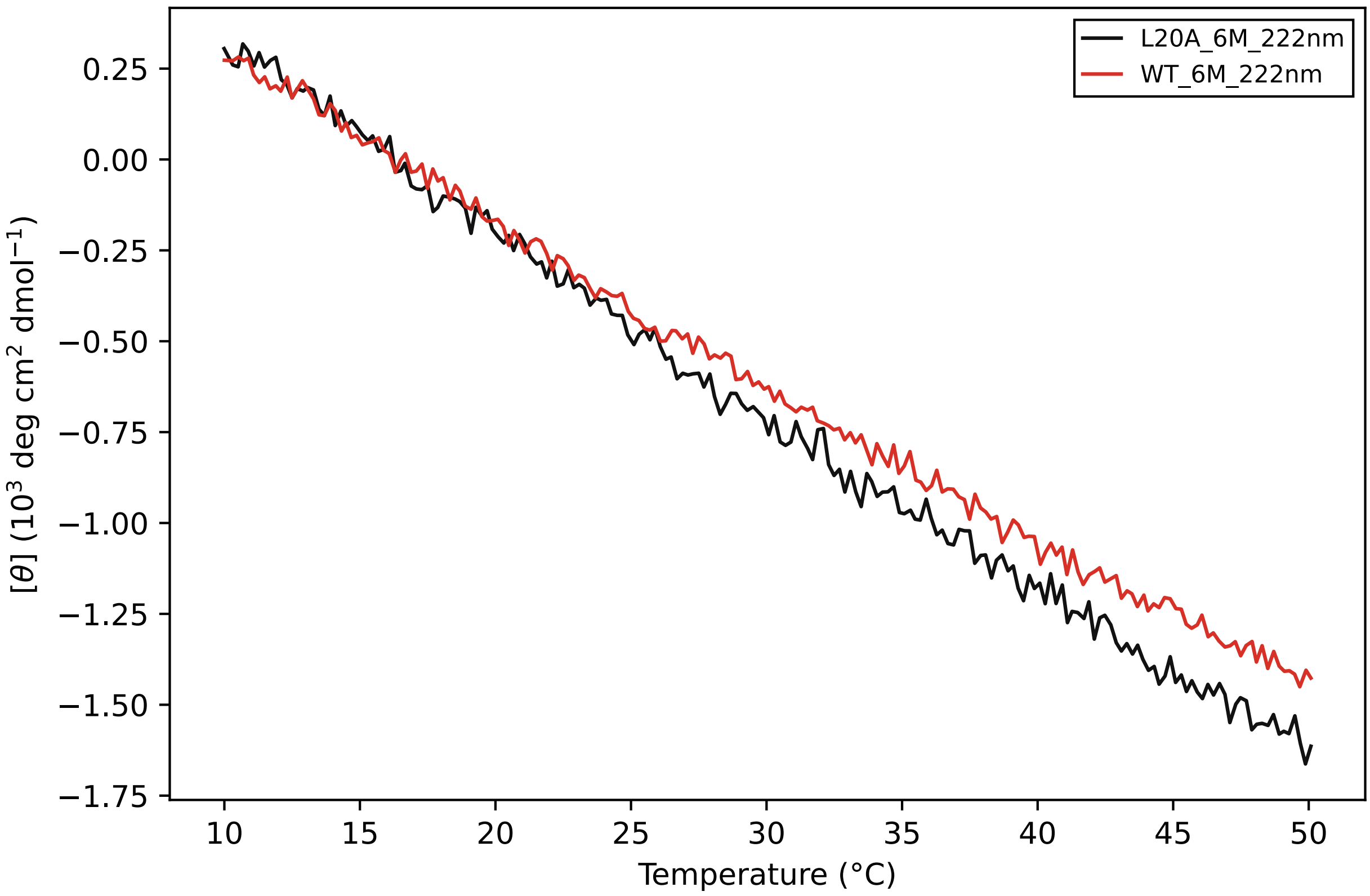

Supplementary Figure S3.

Temperature dependence of the ellipticity at 222 nm for WT (red) and L20A (black) BDPA in 6.0 M GdmCl, monitored during heating from 10 to 50 °C. Measurements were performed in 0.10 M NaCl–20 mM HCOONa buffer in 90% D_2O/10% H_2O at pH* 3.4.

Supplementary Table S2.

	WT	L20A
Residue	helical propensity	helical propensity
G -2	0	0
S -1	0.02	0.02
H0	0.11	0.11
M1	0.13	0.13
A2	0.18	0.18
D3	0.18	0.18
N4	0.24	0.24
K5	0.77	0.73
F6	0.73	0.69
N7	0.78	0.73
K8	1.1	1.03
E9	1.33	1.22
Q10	1.54	1.4
Q11	1.61	1.45
N12	1.7	1.52
A13	3.25	2.89
F14	3.34	2.95
Y15	3.5	3.07
E16	3.49	3.05
I17	3.49	3.05
L18	3.4	2.97
H19	1.86	1.42
L(A)20	0.01	0
P21	0.03	0.03
N22	0.04	0.04
L23	0.4	0.41
N24	0.45	0.46
E25	2.03	2.03
E26	2.1	2.11
Q27	1.97	1.98
R28	1.92	1.93

N29	1.27	1.27
G30	0.93	0.93
F31	0.56	0.56
I32	0.69	0.69
Q33	0.85	0.85
S34	0.83	0.83
L35	0.79	0.79
K36	0.67	0.67
D37	0.07	0.07
D38	0.01	0.01
P39	0.13	0.13
S40	0.22	0.22
Q41	1.69	1.69
S42	1.98	1.98
A43	4.74	4.74
N44	4.97	4.97
L45	8.12	8.12
L46	8.91	8.9
A47	10.32	10.32
E48	10.88	10.88
A49	10.86	10.85
K50	9.36	9.35
K51	7.7	7.69
L52	6.78	6.78
N53	5.45	5.45
D54	1.53	1.53
A55	1.07	1.07
Q56	0.16	0.16
A57	0	0
P58	0	0
K59	0	0

Residue-specific helical propensities of the wild type and the L20A mutant were predicted using Agadir 3 without terminal protection effects, using the same temperature, ionic strength, and pH as in the CD experiments [3].

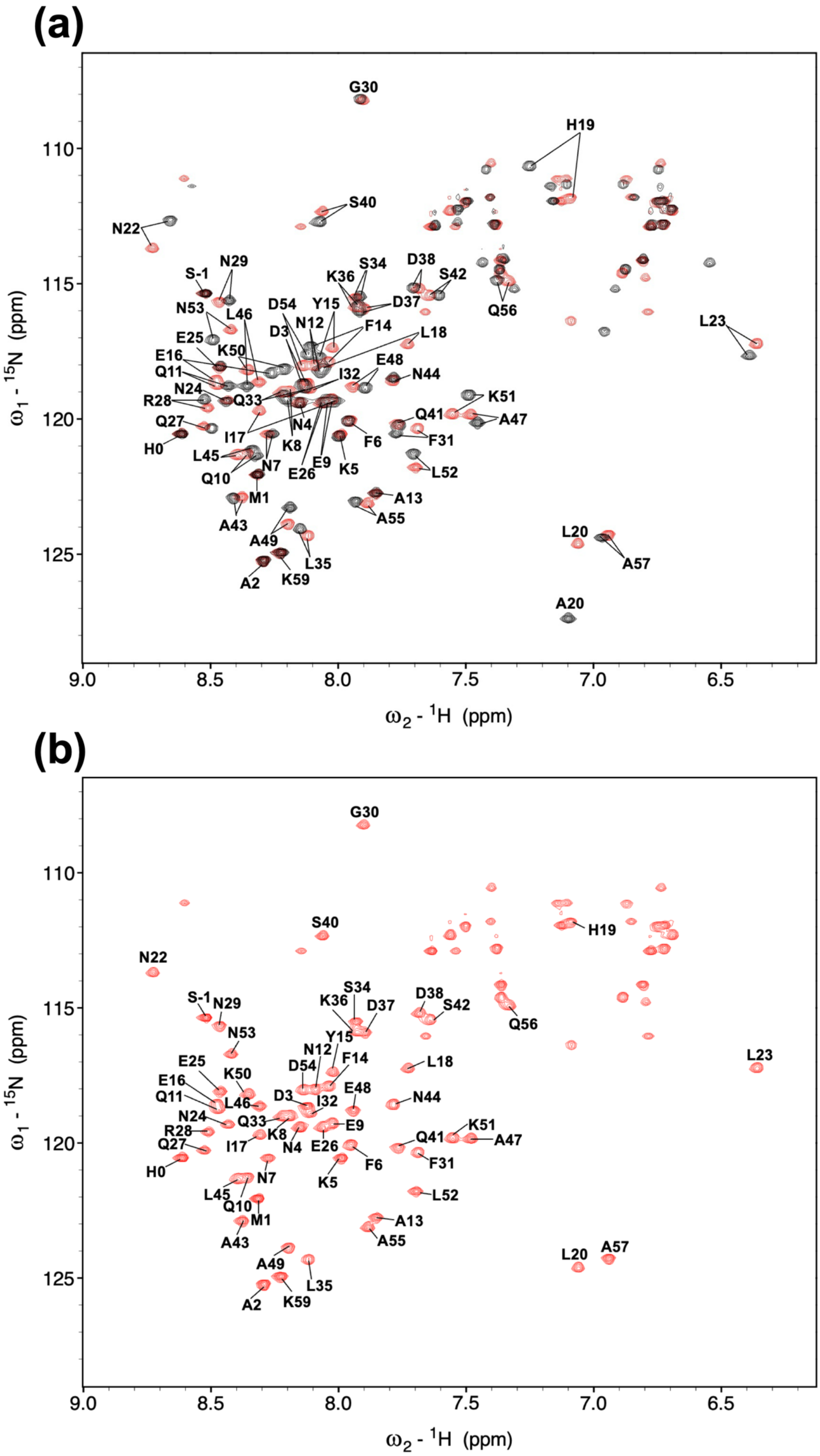
(a)
G30
H19
S40
N22
S-1
N29
N53
L46
K36
S34
D38
S42
D54
Y15
D37
Q56
N12
F14
L18
L23
D3
E25
E16
K50
E48
Q11
N44
N24
I32
R28
Q33
K51
A47
Q41
Q27
N4
F31
I17
H0
K8
F6
L45
E9
N7
K5
L52
Q10
E26
M1
A13
A55
A43
A49
L20
L35
A57
A2
K59
A20
110
115
120
125
9.0
8.5
8.0
7.5
7.0
6.5
ω1 - 15N (ppm)
ω2 - 1H (ppm)
(b)
G30
S40
H19
N22
S-1
N29
S34
K36
D37
D38
S42
N53
Q56
Y15
N12
F14
L18
L23
D54
E25
K50
E16
Q11
L46
D3
I32
E48
N44
N24
Q33
E9
R28
K8
K51
Q41
A47
Q27
I17
N4
E26
F31
H0
F6
N7
K5
L45
Q10
L52
M1
A13
A43
A55
A57
L20
A49
L35
A2
K59
110
115
120
125
9.0
8.5
8.0
7.5
7.0
6.5
ω1 - 15N (ppm)
ω2 - 1H (ppm)

Supplementary Figure S4.

(a) Overlay of 2D ^{1}H–^{15}N HSQC spectra of wild-type (red) and L20A (black) BDPA in the N state. (b) 2D ^{1}H–^{15}N HSQC spectra of wild-type. Measurements were conducted in 0.10 M NaCl and 20 mM sodium formate buffer in 10% D_2O at pH 3.4 at 15.0 °C.

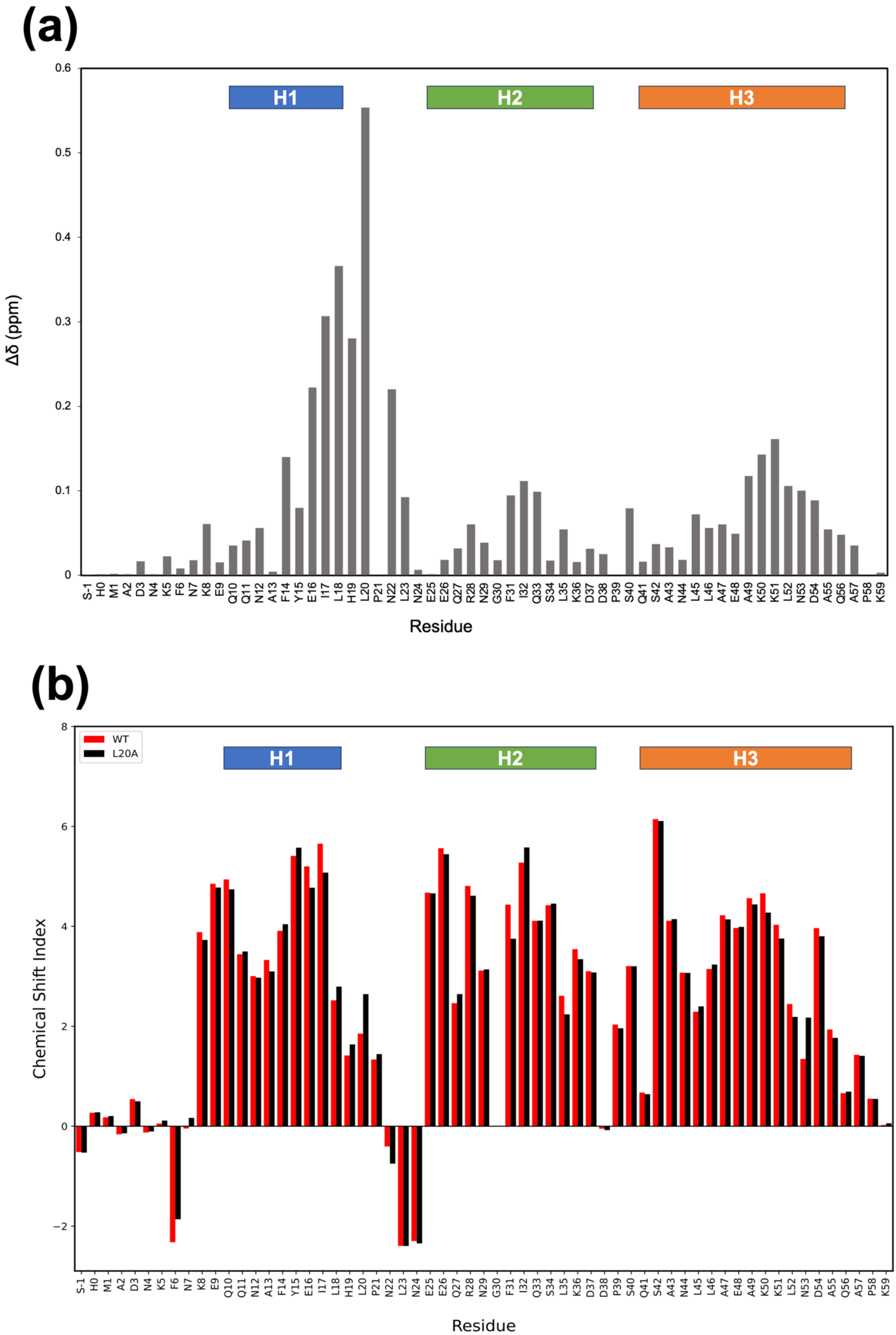
(a)
H1
H2
H3
Δδ (ppm)
Residue
(b)
WT
L20A
H1
H2
H3
Chemical Shift Index
Residue

Supplementary Figure S5.

(a) Chemical shift perturbation between the wild-type and the L20A mutant in the N state. (b) Residue-wise chemical shift index of the wild-type (red) and the L20A mutant (black) in the N state. Helices H1, H2, and H3 are indicated using the same color scheme as in Fig. 1.

(a)

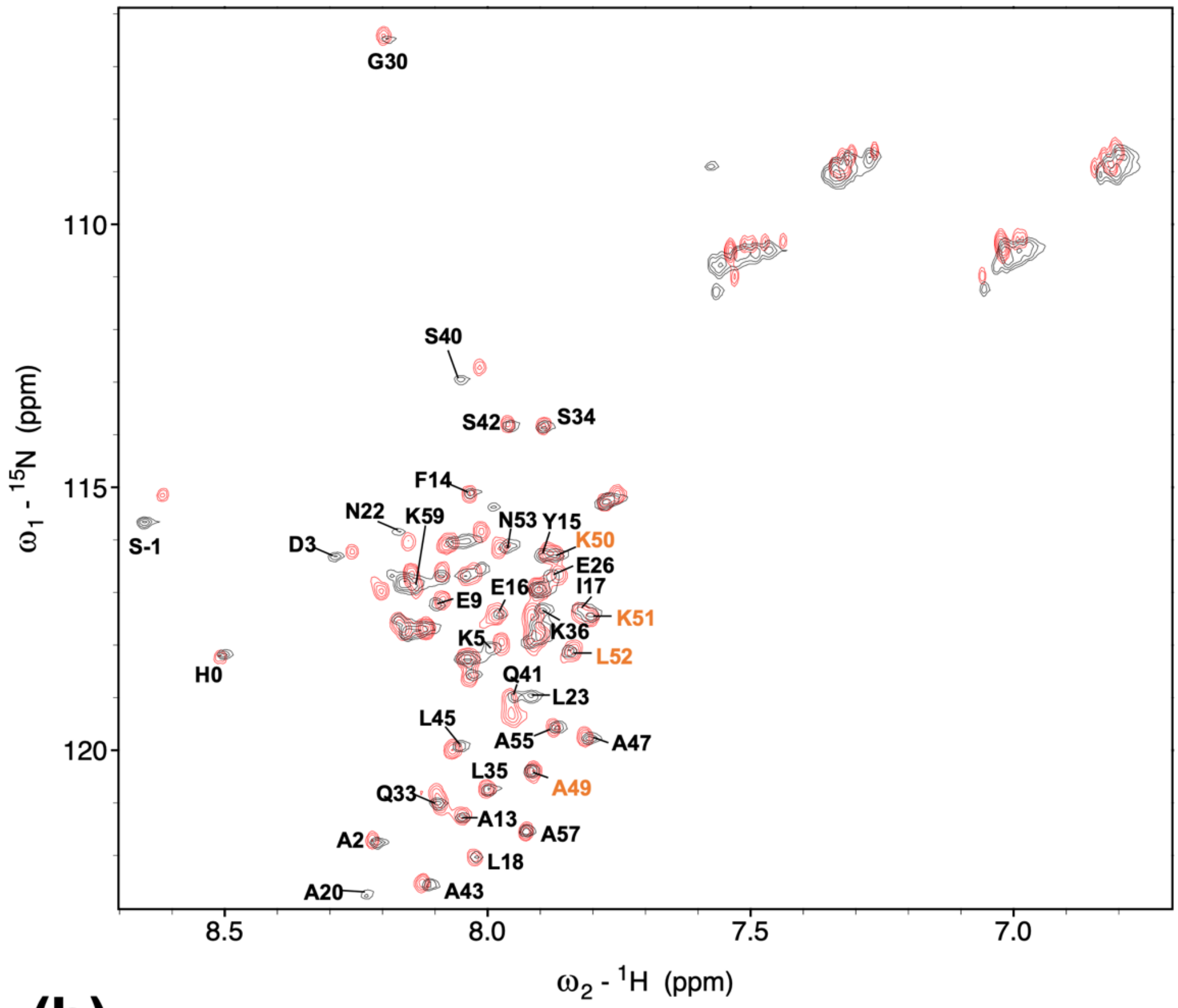
G30
S40
S42
S34
F14
N22
K59
N53
Y15
K50
S-1
D3
E26
E16
I17
E9
K51
K36
K5
L52
H0
Q41
L23
L45
A55
A47
L35
A49
Q33
A13
A57
A2
L18
A20
A43
110
115
120
8.5
8.0
7.5
7.0
ω1 - 15N (ppm)
ω2 - 1H (ppm)

(b)

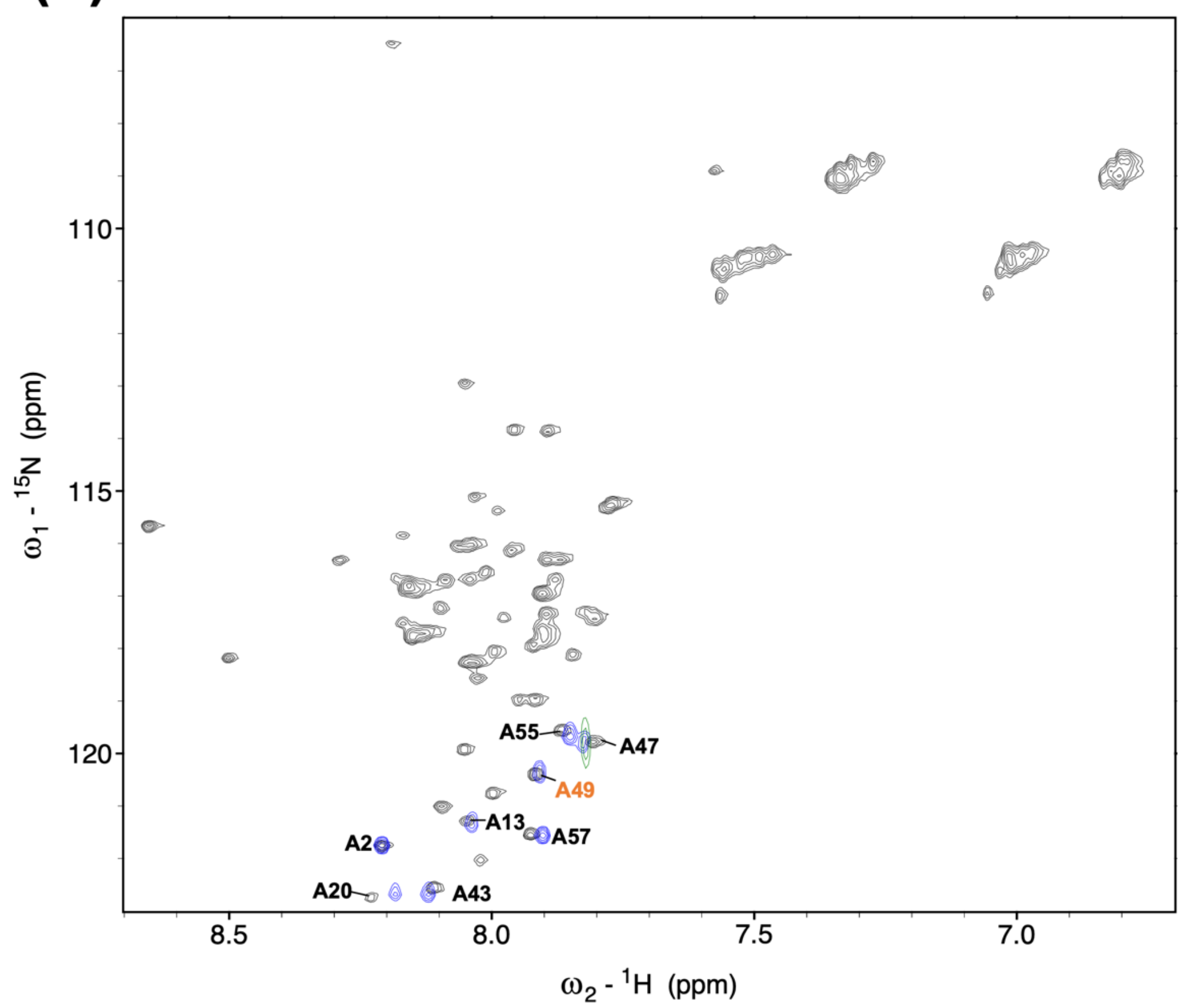
A55
A47
A49
A13
A57
A2
A20
A43
110
115
120
8.5
8.0
7.5
7.0
ω1 - 15N (ppm)
ω2 - 1H (ppm)

63

Supplementary Figure S6.

(a) Overlay of 2D ^{1}H–^{15}N HSQC spectra of wild-type (red) and L20A (black) BDPA in DMSO/H2O solution (94.5% DMSO-d6 / 5.0% H2O / 0.5% DCA-d2, pH* 5.4) at 25 °C. The close correspondence of peak positions is consistent with similarly unstructured conformations under these DMSO-based conditions and supports transfer of backbone assignments from wild-type BDPA to the L20A mutant. Residues analyzed in the H/D-exchange experiments are annotated. (b) Spectral comparison used for assignment confirmation of selectively labeled L20A. Overlay of the ^{15}N-labeled L20A ^{1}H–^{15}N HSQC spectrum (black) with the ^{1}H–^{15}N HSQC (blue) and HNCO (green) spectra of the ^{13}C-leucine/^{15}N-alanine selectively labeled L20A sample under the same DMSO/H2O conditions. Residues 49–52 are depicted in orange in both panels (a) and (b).

Supplementary Table S3. H/D-exchange parameters of backbone NH groups of wild-type and L20A mutant BDPA in 6.0 M GdmCl (pH* 3.2–3.4, 15 °C), and the secondary structures in the N state of wild-type BDPA (PDB code: 1BDD)

	Wild-type BDPA[a]			L20A Mutant[b]			
Residue	$k_{obs} \times 10^2$ (min^{-1})	$k_{int} \times 10^2$ (min^{-1})	P	$k_{obs} \times 10^2$ (min^{-1})	$k_{int} \times 10^2$ (min^{-1})	P	Secondary structure[c]
A2		8.52		15.60±4,39	7.80	0.66±0.13	Coil
D3		27.88		39.31±18.43	25.87	0.95±0.25	Coil
N4		67.93			63.11		Coil
K5	5.15±0.08	12.49	2.42±0.04	7.42±0.38	11.41	1.53±0.06	Coil
F6		4.99			4.56		Turn
N7		23.20			21.18		Turn
K8		12.49			11.41		Turn
E9	4.02±0.17	14.36	3.58±0.15	5.95±0.49	13.18	2.17±0.09	Turn
Q10		17.46			16.05		α-Helix
Q11		11.92			10.89		α-Helix
N12		32.02			29.24		α-Helix
A13	4.72±0.17	13.80	2.92±0.16	7.91±1.10	12.62	1.60±0.08	α-Helix
F14	2.61±0.13	3.82	1.46±0.07	3.71±0.31	3.50	0.77±0.06	α-Helix
Y15	2.01±0.05	4.06	2.02±0.06	3.02±0.47	3.71	1.07±0.14	α-Helix
E16	3.26±0.08	12.22	3.75±0.10	4.68±0.32	11.22	2.11±0.33	α-Helix
I17	1.22±0.05	2.84	2.34±0.10	1.42±0.18	2.61	1.31±0.22	α-Helix
L18	1.03±0.07	1.03	0.99±0.07	1.48±0.21	0.94	0.53±0.05	α-Helix
H19		25.43			23.22		Coil
L(A)20		11.63		15.81±2.76	40.37	2.81±0.17	Turn
P21	n.a.	n.a.	n.a.	n.a.	n.a.	n.a.	Turn
N22	8.32±0.43	25.40[d]	3.05±0.16	14.36±2.56	23.19[d]	1.69±0.07	Turn
L23		3.63		4.65±0.23	3.32	0.71±0.04	Turn
N24	5.18±0.54	12.49	2.41±0.25		11.41		Coil
E25		22.76			20.88		α-Helix
E26		25.29			23.34		α-Helix
Q27		17.46			16.05		α-Helix
R28		12.48			11.39		α-Helix

N29	13.36±1.09	33.53	2.51±0.21		30.61		α-Helix
G30	8.11±0.42	12.83	1.58±0.08	13.36±2.12	11.73	0.83±0.05	α-Helix
F31		5.65			5.19		α-Helix
I32		1.41			1.29		α-Helix
Q33		4.43		4.38±0.39	4.05	0.87±0.06	α-Helix
S34	7.42±0.18	24.31	3.28±0.08	10.48±0.88	22.20	2.12±0.10	α-Helix
L35	2.58±0.11	3.45	1.34±0.06	3.78±0.22	3.15	0.80±0.07	α-Helix
K36		3.72		3.32±0.44	3.40	0.93±0.06	α-Helix
D37		36.50			33.84		α-Helix
D38		93.07			87.78		Turn
P39	n.a.	n.a.	n.a.	n.a.	n.a.	n.a.	Turn
S40	8.36±1.00	19.28[d]	2.31±0.28	15.90±2.90	17.61[d]	1.19±0.04	Turn
Q41		14.99		10.11±0.94	13.69	1.06±0.08	α-Helix
S42	9.53±0.45	24.31	2.55±0.12	13.02±1.05	22.20	1.64±0.08	α-Helix
A43	6.15±0.12	13.12	2.13±0.04	9.08±0.81	11.99	1.28±0.03	α-Helix
N44	9.99±0.82	20.24	2.03±0.17		18.49		α-Helix
L45	1.86±0.11	3.63	1.95±0.11	2.82±0.22	3.32	1.17±0.09	α-Helix
L46		1.10			1.01		α-Helix
A47	1.94±0.12	4.18	2.15±0.13	2.83±0.42	3.84	1.31±0.03	α-Helix
E48	3.84±0.27	10.93	2.85±0.20		10.03		α-Helix
A49	2.93±0.16	15.30	5.23±0.28	4.37±0.41	14.08	3.30±0.21	α-Helix
K50		6.01		3.66±0.64	5.50	1.66±0.14	α-Helix
K51	1.50±0.15	7.88	5.25±0.53	3.45±0.24	7.20	2.16±0.23	α-Helix
L52	1.54±0.08	2.29	1.49±0.08	2.32±0.18	2.10	0.87±0.06	α-Helix
N53	7.53±0.31	12.49	1.66±0.07	10.11±1.25	11.41	1.23±0.13	α-Helix
D54		57.82			53.61		α-Helix
A55	5.50±0.60	22.23	4.06±0.44	10.12±1.57	20.68	2.24±0.11	α-Helix
Q56	4.00±0.12	7.57	1.89±0.06		6.92		α-Helix
A57	2.51±0.13	10.46	4.17±0.21	5.19±1.22	9.57	2.17±0.16	Coil
P58	n.a.	n.a.	n.a.	n.a.	n.a.	n.a.	Coil

K59	2.74±0.18	3.63[d]	1.34±0.09		3.64[d]		Coil

[a]The k_{obs}, k_{int} and P values were taken from Yanaka *et al.*, (2023) [4]. To accurately show the error bars in Figure 4, the P values are displayed to two decimal places. The k_{obs} values were determined by three independent H/D-exchange experiments at pH* 3.4, the average values and the standard errors are shown for k_{obs} and P, and the k_{int} values are those at pH* 3.40.

[b]Three to four independent H/D-exchange experiments were performed for L20A at pH* 3.2–3.4. The k_{int} values at pH* 3.4 are approximately 1.5 times those at pH* 3.2, but the P values are expected to be constant in this pH* range. To compar with the values for the wild-type protein, the k_{obs} values given are those experimentally observed at pH* 3.36, the errors shown are fitting error estimates, and the k_{int} values are those at pH* 3.36. The P values were thus evaluated by the 3–4 independent exchange experiments, and the average values and the standard errors are given in the table.

[c]Secondary structure in the N state of wild-type BDPA (PDB code: 1BDD) determined by the program STRIDE [5].

[d]The k_{int} values of the residues (N22, S40, and K59), following the proline residues, depend on the *cis–trans* isomer state of the proline residue [6], and the k_{int} is given by:

$$k_{int} = 0.2\ k_{int,cis} + 0.8\ k_{int,trans}$$

where $k_{int,cis}$ and $k_{int,trans}$ are the intrinsic exchange rate constants of the *cis* and *trans* isomers, respectively, and we have assumed that 80% of each proline residue is in the *trans* form in 6.0 M GdmCl.

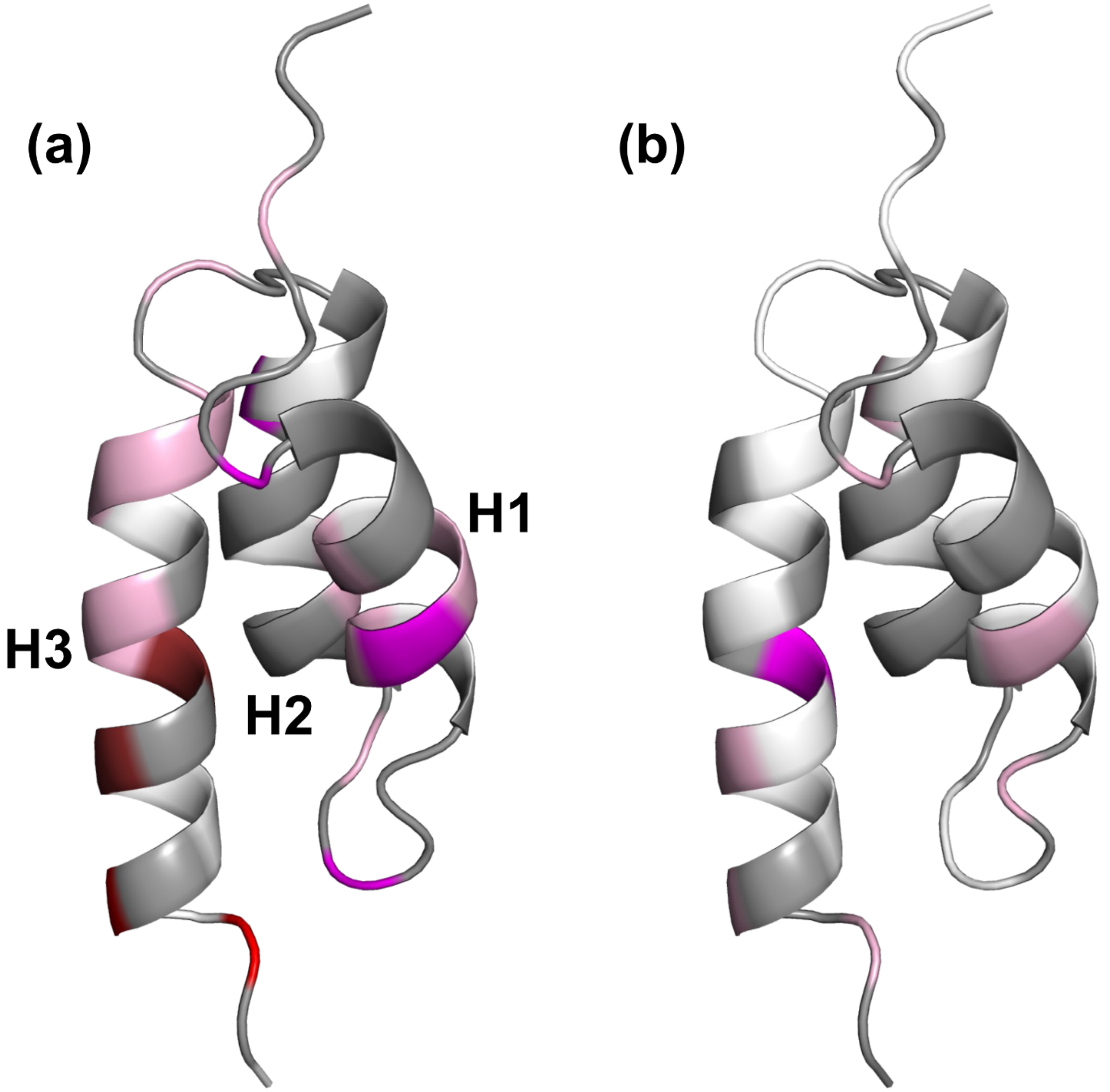

Supplementary Figure S7.

Structural mapping of H/D-exchange protection in wild-type and L20A BDPA. The protection factors listed in Table S3 were mapped onto the native structure of BDPA (PDB ID: 1BDD) for (a) wild-type BDPA and (b) the L20A mutant. The residues with *P* values larger than 2.0, 3.0, 4.0, and 5.0 are colored light magenta, magenta, red, and dark red, respectively, whereas residues for which *P* values were not determined are shown in gray. The two structures are displayed in the same orientation to facilitate comparison. The mapping highlights the marked reduction in protection in the L20A mutant, particularly in the C-terminal region of helix H3.

References

1. Nagy, G., Igaev, M., Jones, N. C., Hoffmann, S. V. & Grubmüller, H. SESCA: Predicting circular dichroism spectra from protein molecular structures. *J. Chem. Theory Comput.* **15**, 5087–5102 (2019).
2. Nagy, G., Hoffmann, S. V., Jones, N. C. & Grubmüller, H. Reference data set for circular dichroism spectroscopy comprised of validated intrinsically disordered protein models. *Appl. Spectrosc.* **78**, 897–911 (2024).
3. Muñoz, V. & Serrano, L. Elucidating the folding problem of helical peptides using empirical parameters. *Nat. Struct. Biol.* **1**, 399–409 (1994).
4. Yanaka, S., Yagi-Utsumi, M., Kato, K. & Kuwajima, K. The B domain of protein A retains residual structures in 6 M guanidinium chloride as revealed by hydrogen/deuterium-exchange NMR spectroscopy. *Protein Sci.* **32**, e4569 (2023).
5. Heinig, M. & Frishman, D. STRIDE: a web server for secondary structure assignment from known atomic coordinates of proteins. *Nucleic Acids Res.* **32**, W500-2 (2004).
6. Bai, Y., Milne, J. S., Mayne, L. & Englander, S. W. Primary structure effects on peptide group hydrogen exchange. *Proteins* **17**, 75–86 (1993).
